# Intravitreal Bevacizumab Alone or Combined with Macular Laser Photocoagulation for Recurrent or Persistent Macular Edema Secondary to Branch Retinal Vein Occlusion

**DOI:** 10.1155/2014/173084

**Published:** 2014-07-07

**Authors:** Takafumi Hirashima, Tomoyuki Chihara, Toshitaka Bun, Takao Utsumi, Miou Hirose, Hideyasu Oh

**Affiliations:** Department of Ophthalmology, Hyogo Prefectural Amagasaki Hospital, 1-1-1 Higashi-daimotsu-cho, Amagasaki, Hyogo 660-0828, Japan

## Abstract

*Background*. To evaluate the efficacy of intravitreal bevacizumab (IVB) injection with or without macular laser photocoagulation (MLP) for recurrent or persistent macular edema (ME) secondary to branch retinal vein occlusion (BRVO). *Methods*. Thirty-four eyes underwent IVB injection for ME secondary to BRVO as a primary treatment. Twenty of the 34 eyes experienced recurrent or persistent ME after the first IVB. Nine of the 20 eyes (Group 1) were retreated with IVB combined with MLP. The remaining 11 eyes (Group 2) were retreated with IVB alone. *Results*. In Group 1, the postoperative best corrected visual acuity (BCVA) improved compared with the preoperative value at all follow-up visits, although no statistically significant improvement was observed at 6 months. In contrast, BCVA significantly improved from 0.53 to 0.40 at 6 months (*P* < 0.05) in Group 2. *Conclusion*. Combined therapy tended to have a smaller effect on visual acuity compared with IVB monotherapy.

## 1. Introduction

Branch retinal vein occlusion (BRVO) is considered the second most common retinal vascular disease, the frequency of which is surpassed only by diabetic retinopathy [1–3]. Macular edema (ME) from BRVO is a frequent cause of visual acuity (VA) loss [3, 4]. The gold standard for treating ME associated with BRVO has been considered to be grid laser photocoagulation since the Branch Vein Occlusion Study Group reported in 1984 that patients with ME associated with BRVO and a VA of 20/40 or less showed a significant visual benefit compared with an untreated control group [5, 6].

In 1994, Aiello et al. showed that intravitreal levels of the vascular endothelial derived growth factor protein (VEGF) are significantly increased after BRVO and this leads to dysfunction of the endothelial blood-retinal barrier and to increased vascular permeability, resulting in ME [7]. Since the first report on the efficacy of intravitreal bevacizumab (IVB) in a patient with ME secondary to CRVO in 2005 [8], a number of case series have shown that it has promising effects, with rapid reduction in foveal thickness and improvement in VA [9–12]. However, the main limitations of this treatment are its short-term effects and a high reported rate of ME recurrence [8, 13]. Some formats of retreatment were previously described not only in the era of BRVOS study [5] but also in the recent BRAVO study [14]. However, there are no established retreatment protocols that delineate the criteria and time intervals for retreatment. In this retrospective study, we report the efficacy of retreatment using IVB alone or IVB combined with macular laser photocoagulation (MLP).

## 2. Patients and Methods

### 2.1. Subjects

Thirty-four eyes of 34 patients that were diagnosed with ME secondary to BRVO and received IVB as the initial treatment at the Amagasaki Hospital from February 2010 through August 2012 were retrospectively reviewed. At each visit, all participants underwent a comprehensive ophthalmic examination including measurement of best corrected visual acuity (BCVA) with the use of a 5 m Landolt chart, slit-lamp biomicroscopy, intraocular pressure measurement, dilated stereoscopic fundus examination, and SD-OCT imaging with a macular cube 256 × 128 scan protocol (Cirrus HD-OCT; Carl Zeiss Meditec, Dublin, CA, USA). The tomographic parameter analyzed in this study was the central subfield thickness (CST), which was calculated as the average retinal thickness within a circle of 1 mm radius centered on the fovea. At the initial visit for each patient, fluorescein angiography was performed with a confocal laser scanning system (HRA-2; Heidelberg Engineering, Heidelberg, Germany).

Eyes with other ocular diseases (e.g., epiretinal membrane, glaucoma, or proliferative diabetic retinopathy) were not recruited for this study.

After initial IVB, 20 eyes had recurrent or persistent ME. The recurrent ME are the cases in which the CST resolved to no more than 250 *μ*m at least once after the first IVB and then increased to over 250 *μ*m. Persistent ME cases were defined as those in which the CST also did decrease after the first IVB, but was still above 250 *μ*m. During the follow-up of these 20 eyes, 9 eyes were retreated with IVB combined with MLP (Group 1) and 11 eyes were treated with IVB alone (Group 2). Retreatments were only performed if OCT showed persistent or recurrent ME. In cases that we consider retreatment is needed, we explained to all of them about the combined therapy to the patients as a treatment option and performed it to only those we could obtain informed consent. The decision was made every time when recurrence or persistence of ME was observed, so actually, there were 3 patients who refused the combined therapy at the initial recurrence or persistence but agreed at later visits. This study was approved by the Institutional Review Board and Ethics Committee and conformed to the Declaration of Helsinki. Informed consent was obtained from all patients.

### 2.2. Intravitreal Bevacizumab Injection

Bevacizumab (Avastin; Genentech, South San Francisco, CA, USA), 1.25 mg in a 0.05 mL total volume, was injected intravitreally via the pars plana. Following the injection, retinal perfusion was controlled. All injections were performed in a sterile fashion and prophylactic topical antibiotics were applied for 2 weeks after the injection.

All patients were informed about the potential side effects of bevacizumab treatment, and those with contraindications for IVB (e.g., acute ocular infection, recent history of stroke or myocardial infarction, uncontrolled hypertension, uncompensated renal insufficiency, allergy to bevacizumab, or pregnancy) were not included in this study.

### 2.3. Macular Laser Photocoagulation

We basically selected combined IVB/MLP therapy when the leaking area was visible based on the fluorescein angiogram. However, when we could not obtain informed consent from those patients even after a full explanation of MLP, we did not perform MLP and selected IVB monotherapy for patients even with the leaking area. A multicolor diode laser (MC-300; Nidek, Gamagori, Japan) with settings of 561 nm in wavelength and a 100 *μ*m spot size for 0.1 second together with sufficient power (median, 100 mW; range, 70–140 mW) was used to produce a burn detectable at the level of the retinal pigment epithelium. MLP was performed within 3 weeks after the precedent IVB and areas with residual retinal edema (except the foveal avascular area) and/or microaneurysms were treated. Two doctors (Hideyasu Oh and Tomoyuki Chihara) performed the MLP treatment.

### 2.4. Follow-Up

The 20 patients had at least 6 months' follow-up after the second IVB. We followed up at month 1 and month 3 and every 3 months thereafter after the second IVB. When retreatment was performed, patients were additionally followed up at month 1 after the retreatment, on top of the above described follow-up schedule. In all visits of all patients, OCT was performed to ascertain recurrence/persistence of ME. Subsequent FA was performed when enlargement of nonperfusion area was suspected, especially when sheathed vessels became evident after the resolution of initial retinal hemorrhages.

### 2.5. Statistics

Statistical analysis was performed with SPSS (SPSS version 20.0; Chicago, IL, USA). BCVA was converted to the logarithm of the minimum angle of resolution (logMAR) before analysis. The statistical significance of differences between values for Group 1 and Group 2 was evaluated with a Mann-Whitney test. *P* values of <0.05 were considered statistically significant.

## 3. Results

### 3.1. Patient Characteristics

Subject characteristics are shown in Table 1. The mean age of the patients was 69.0 ± 9.9 years (range 38–83 years). The mean follow-up was 15.8 ± 4.8 months (range 10–54 months). The mean number of IVB treatments during 12 months' follow-up was 2.50 ± 0.89. The mean duration between the onset of BRVO and the first IVB was 19.6 ± 7.2 days in the 34 patients and 18.8 ± 5.2 in the 20 patients, respectively. There was also no significant difference in the mean duration *d* between Group 1 and Group 2 (19.8 ± 5.7 days in Group 1 versus 17.7 ± 4.5 in Group 2; *P* = 0.37). The mean duration between the initial IVB and the second IVB in those 20 patients was 3.5 ± 0.9 months. There was also no significant difference in this duration between the two groups (3.3 ± 0.7 months in Group 1 versus 3.6 ± 1.2 in Group 2; *P* = 0.49).

### 3.2. Recurrent or Persistent ME

After the initial IVB treatment (termed “baseline”), 18 eyes and 2 eyes had recurrent and persistent ME, respectively. No specific nonperfusion area for persistent ME was found.

### 3.3. Visual Acuity Outcome

Just before the second IVB treatment, the mean BCVA level was 0.56 ± 0.33. At 1 month and 3 months after the second IVB treatment, the mean BCVA had improved to 0.40 ± 0.29 and 0.37 ± 0.27, respectively. At 6 months, the BCVA was still maintained at 0.40 ± 0.25, which was significantly better compared with the baseline BCVA (*P* < 0.05) (Table 2).

**Figure 1:**
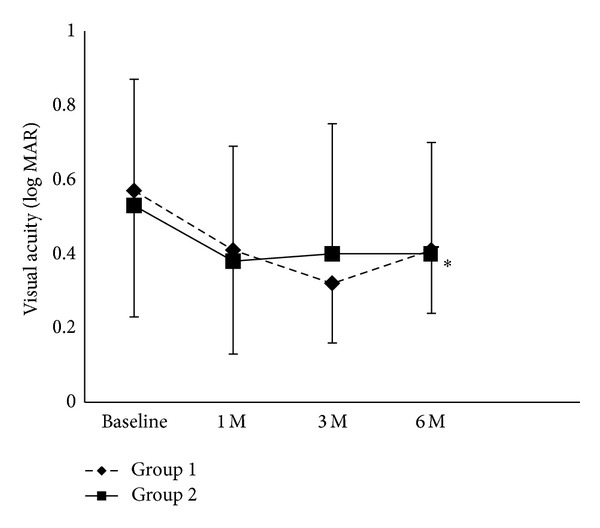
Best corrected visual acuity in logMAR for both eyes of Group 1 and Group 2. Notes: *P < 0.05, compared with the respective baseline value (just before the second intravitreal bevacizumab treatment).

In Group 1, the BCVA improved from 0.57 to 0.41 at 1 month, 0.32 at 3 months, and 0.41 at 6 months after the second IVB treatment, but the change was not statistically significant at 6 months (Figure 1). In contrast, BCVA significantly improved from 0.53 to 0.40 at 6 months (*P* < 0.05) in Group 2 (Figure 1).

### 3.4. Imaging Outcome

The mean baseline CST was 478.1 ± 192.9 *μ*m. At 1 month, 3 months, and 6 months after the second IVB treatment, the mean CST decreased to 296.1 ± 108.9 *μ*m, 340.0 ± 98.7 *μ*m, and 305.0 ± 127.9 *μ*m (*P* < 0.01 at 6 months versus baseline), respectively (Table 2).

**Figure 2:**
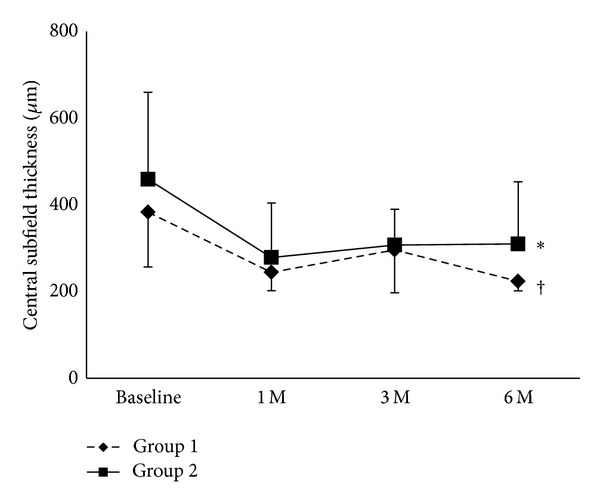
Central subfield thickness assessed with spectral domain optical coherence tomography in both eyes of Group 1 and Group 2. Notes: *P < 0.05 and ^†^ *P* < 0.01, compared with the respective baseline value (just before the second intravitreal bevacizumab treatment).

In Group 1, the CST significantly decreased from 431.2 *μ*m to 274.9 *μ*m at 1 month, 332.9 *μ*m at 3 months, and 251.7 *μ*m at 6 months (*P* < 0.01 at 6 months versus baseline; Figure 2). In Group 2, significant reduction of CST compared with the baseline value was also observed at 6 months (*P* < 0.05; Figure 2).

### 3.5. Comparison of BCVA, CST, and the Number of IVB Treatments between Group 1 and Group 2

As to the changes of BCVA, the average changes from the baseline to 6 months in Group 1 and Group 2 were 0.15 and 0.13, respectively. However, there was no significant difference between the groups. Similarly, no significant difference was observed regarding the changes of CST from the baseline to 6 months between groups (Figures 1 and 2). The mean number of IVB treatments in Group 1 was 2.44 ± 1.24 during the 12-month follow-up period, compared with 2.55 ± 0.52 in Group 2 (*P* = NS; Table 1).

### 3.6. MLP Therapy

The mean number of MLP treatments was 1.22 during the follow-up. Initial MLP was performed after the second IVB treatment in 6 patients (67%) and after the third IVB treatment in the remaining 3 patients (33%). Seven patients (78%) underwent MLP once and the other two patients (22%) twice. In the two patients that underwent MLP two times during the follow-up, ME resolved after the initial laser treatment and recurred after 2 months and 5 months. After the second laser treatment, no recurrence was observed in these two patients for at least 15 months.

### 3.7. Fluorescein Angiography

The disc area of nonperfusion area evaluated on FA at baseline was 8.3 ± 5.6 in Group 1 and 9.2 ± 3.9 in Group 2 (*P* = 0.69). There was no significant difference in cases that required panretinal coagulation between the two groups. (5 of 9 eyes in Group 1 versus 7 of 11 eyes in Group 2; *P* = 0.71).

As to the type of angiographic type, all of those 20 patients had cystoid macular edema with visible microaneurysms (11 eyes) or without (9 eyes). There was no significant difference in the percentage of cases with microaneurysms between groups (7 of 9 eyes in Group 1 versus 6 of 11 eyes in Group 2; *P* = 0.28, chi-square test), and the baseline BCVA of the 13 eyes with microaneurysms was similar to that of the remaining 7 eyes without microaneurysms (logMAR 0.53 ± 0.34 in BRVO with microaneurysms versus logMAR 0.59 ± 0.33 in BRVO without microaneurysms).

## 4. Discussion 

Recently, several studies have shown the efficacy of anti-VEGF therapy for ME secondary to BRVO [14–16]. Currently, IVB is used to treat ME as a primary treatment in an off-label manner. Although IVB has a rapid and promising effect, the majority of patients require retreatment for ME after IVB because of the short duration of the therapeutic effect [17, 18]. In this study, the rate of retreatment was 58.9%, which is similar to rates reported in previous studies [19, 20]. As to treatment for the recurrence of ME, recent studies have shown that repeated injections with IVB are required to maintain good visual acuity [12, 21]. However, the appropriate method (i.e., IVB, MLP, or combined therapy), criteria, and timing for retreatment remain to be elucidated. Donati et al. reported that IVB combined with MLP as a primary treatment significantly improved mean VA (logMAR) from 0.6 to 0.2 and reduced the mean CST from 386 to 238 one year after the initial treatment [22]. In cases with recurrent ME, Hayashi et al. revealed that combination therapy maintained the mean VA but significantly increased the mean CST after 12 weeks [23]. The aim of our study was to evaluate the efficacy of combination retreatment with IVB and MLP. To the best of our knowledge, this is the first study to compare the efficacy of retreatment with either IVB alone or IVB combined with MLP.

This study showed that retreatment with IVB alone had a substantial effect on both reduction of CST and improvement in VA. In contrast, although the combination retreatment also had a significant effect on reducing CST, its effect on improving VA was limited, which concurs with a recently published report [24]. Although CST tended to be thicker in Group 2, the difference was not statistically significant. Moreover, the relatively thick CST in Group 2 during the follow-up might be at least partly explained by the difference at baseline.

In regard to the number of IVB treatments, previous studies have shown that additional grid laser photocoagulation for recurrent ME in BRVO after IVB treatment may reduce the number of IVB treatments [23, 24]. Because those studies were uncontrolled, were nonrandomized, and involved small case series, it is difficult to draw reliable conclusions. Contrarily, no statistically significant difference in the mean number of IVB treatments needed was observed between the two groups in the current study. Further studies will be required to assess the efficacy of reducing the number of IVB treatments with additional MLP.

In conclusion, treatment with IVB alone seemed to be effective for both reduction of ME and improvement in VA, and combination therapy with MLP did not prove to have additional effects on these parameters. Considering these results and the potential side effects, such as induction of scotoma by grid laser photocoagulation, retreatment with IVB alone might be a feasible option for recurrent or persistent ME secondary to BRVO only. Limitations of the present study were the small sample size and the relatively short follow-up duration of 6 months. Furthermore, although the interval of follow-up we used was similar to some reports like that by Donati et al. [22], it is possible that infrequent visits (compared to monthly follow-up studies) can consequently lead to no difference in the between-group comparison. A prospective randomized patient study is warranted to compare the efficacy of retreatment with IVB alone versus combination retreatment with MLP.

## Figures and Tables

**Table 1:** Patient characteristics by treatment group.

	IVB combined with MLP Group 1 (*n* = 9)	IVB only Group 2 (*n* = 11)	*P* value
Age (years)	70.2 ± 7.16	65.0 ± 13.9	NS^a^
Gender (male/female)	5/4	5/6	NS^b^
Hypertension	5	6	NS^b^
Baseline BCVA (log⁡⁡MAR)	0.57 ± 0.34	0.53 ± 0.34	NS^a^
Baseline CST (*μ*m)	431.2 ± 142.7	516.4 ± 225.3	NS^a^
Number of IVB during 12 months of follow-up	2.44 ± 1.24	2.55 ± 0.52	NS^a^
Number of MLP during 12 months of follow-up	1.22 ± 0.42	0	NA
Duration between onset of BRVO and first IVB (days)	17.7 ± 4.5	19.8 ± 5.7	NS^a^
Duration between first IVB and second IVB (months)	3.3 ± 0.7	3.6 ± 1.1	NS^a^
Follow-up (months)	17.8 ± 3.1	14.5 ± 5.5	NS^a^

BCVA = best corrected visual acuity; CST = central subfield thickness; MLP = macular laser photocoagulation; IVB = intravitreal bevacizumab; log⁡⁡MAR VA = logarithm of the minimal angle of resolution visual acuity; NS = not significant; NA = not available; ^a^Mann-Whitney test; ^b^chi-square test.

**Table 2:** Mean best corrected visual acuity and central subfield thickness at different points.

	BCVA (log⁡⁡MAR)	*P* value	CST (*μ*m)	*P* value
Baseline	0.55 ± 0.33		478.1 ± 192.9	
1 month	0.40 ± 0.29		296.1 ± 108.9	
3 months	0.37 ± 0.27		340.0 ± 98.7	
6 months	0.40 ± 0.25	<0.05∗	305.0 ± 127.9	<0.01^†^

BCVA = best corrected visual acuity; CST = central subfield thickness; log⁡⁡MAR = logarithm of the minimal angle of resolution.

Mean BCVA and mean CST assessed by spectral domain optical coherence tomography at baseline, 1 month, 3 months, and 6 months after the initial retreatment.

*P < 0.05 and ^†^ *P* < 0.01, compared with baseline values (just before the second intravitreal bevacizumab treatment).
